# The increasing burden of asthma acute care in Singapore: an update on 15-year population-level evidence

**DOI:** 10.1186/s12890-023-02797-7

**Published:** 2023-12-12

**Authors:** Laura Huey Mien Lim, Wenjia Chen, Joseph Emil Amegadzie, Hui Fang Lim

**Affiliations:** 1 Saw Swee Hock School of Public Health, National University of Singapore, #10-01, 12 Science Drive 2, 117549 Singapore; 2 Respiratory Evaluation Sciences Program, Collaboration for Outcomes Research and Evaluation, Faculty of Pharmaceutical Sciences, University of British Columbia, 2405 Wesbrook Mall, Vancouver, BC V6T 1Z3 Canada; 3 Division of Respiratory and Critical Care Medicine, Department of Medicine, National University Hospital, National University Health System, NUHS Tower Block, 1E, Kent Ridge Road, 119228 Singapore; 4 Department of Medicine, Yong Loo Lin School of Medicine, National University of Singapore, NUHS Tower Block, 1E, Kent Ridge Road Level 11, 119228 Singapore

**Keywords:** Asthma, COVID-19, Cost of illness

## Abstract

**Background:**

In Singapore, there is currently scarce population-based research informing the recent trends of asthma-related healthcare burdens. In this study, we investigated the past 25-year trends of asthma-related hospitalisations, emergency department (ED) visits and deaths in Singapore and projected the future burdens from 2023 to 2040.

**Methods:**

We acquired annually-measured data from the Singapore Ministry of Health Clinical and National Disease Registry, containing 25-year asthma-related hospitalisation and death rates as well as 15-year ED visit rates. We conducted change-point analysis and generalised linear modelling to identify time intervals with stable trends and estimate asthma-related healthcare utilisation and mortality rates. To project future asthma-related burdens, we developed a probabilistic model which combined projections of future population size with the estimated rate outcomes from the last stable period.

**Results:**

Our results show that the asthma hospitalisation rate in Singapore had remained at approximately 80 episodes per 100,000 from 2003 to 2019 and are likely to grow by 1.7% each year (95% CI: 0.7, 5.0%), leading to a total of 163,633 episodes from 2023 to 2040 which corresponds to an estimated $103,075,820 based on 2022 USD. Besides, Singapore’s asthma-related ED visit rate was 390 per 100,000 in 2019 and is expected to decline by 3.4% each year (95% CI: − 5.8, 0.0%), leading to a total of 208,145 episodes from 2023 to 2040 which corresponds to USD$15,053,795. In contrast, the 2019 asthma-related mortality rate in Singapore was approximately 0.57 per 100,000 and is likely to stay stably low (change per year: -1.3, 95% CI: − 11.0, 4.3%). Between 2023 and 2040, Singapore’s estimated total number of asthma-related deaths is 638 episodes.

**Conclusions:**

Currently, the burden of asthma acute care in Singapore is high; Singapore’s asthma-related hospitalisation and ED visit rates are relatively higher than those of other developed economies, and its asthma admission rate is expected to increase significantly over time, possibly indicating excess resource use for asthma. The established national asthma programme in Singapore, together with recent efforts in reinforcing primary care at the national level, provides opportunities to reduce avoidable asthma admissions.

**Supplementary Information:**

The online version contains supplementary material available at 10.1186/s12890-023-02797-7.

## Background

In 2019, asthma affected approximately 262 million people and resulted in 455,000 deaths [1]. Asthma is among the top contributors to the economic burden of chronic diseases [2, 3]. Despite the decline in asthma hospitalisation rates in many countries in The Organisation for Economic Cooperation and Development (OECD) between 2009 and 2019 including Canada (16.8 to 13.7 per 100,000) and Australia (69.8 to 63.1 per 100,000) [4], inpatient expenditures is still the second most expensive component of asthma costs in many jurisdiction, trailing only medication expenses [5].

In Singapore, asthma affects 20% of children and 5% of adults [6]. The annual economic burden of asthma is estimated to be SGD$2.09 billion [7], and asthma-related hospitalisation accounts for a huge burden. However, there is scarce up-to-date population-based evidence on the current and future preventable burden of asthma in Singapore. While the Singapore National Asthma Programme (SNAP) has continuously strived to address the high burden of asthma in Singapore since its establishment in 2001 through improved preventive treatment (with proven success [8]), the population-level burden of asthma in Singapore has not been re-evaluated since 2003 [9], and the most recent asthma economic burden study was based on a survey of a relatively small random sample of 300 asthmatic adults and 221 parents of asthmatic children [7]. As timely and accurate population-based evidence of the burden of asthma is crucial for policymaking and resource allocation at the national level, we undertake this present study to estimate the recent trends and project the future healthcare burden of asthma in Singapore in terms of hospitalisation, emergency department (ED) attendances and mortality.

## Methods

We performed analyses on nationwide aggregate-level data on asthma-related acute care and mortality obtained from the Singapore Ministry of Health Clinical and National Disease Registry. The data included population-level annual asthma admission rates and age- and sex-standardised mortality rates (1994-2020), and ED attendance rates (2005-2020). The study subjects involved were patients, of all ages, with asthma as the final diagnosis during a hospitalisation episode or an ED visit, or who died due to asthma. Asthma-related hospitalisations, ED visits and mortality were defined as inpatient admission episodes, ED visits and death with asthma as the final diagnosis as identified via the International Classification of Diseases (ICD) 9: 493 or ICD 10: J45-J46. Asthma-related hospitalisations are not affected by bronchial thermoplasty (BT) nor injection of biologics; BT is rarely performed (1-2 patients per year) and was not performed between 2020 and 2022 during the height of COVID19, and patients are not hospitalised for biologics injection as it is given in the outpatient setting. Historical data on Singapore’s resident population size (1994-2020) was obtained from the Singapore Department of Statistics (DoS) [10], whereas information on future resident population size (2021-2040) was obtained from “Scenarios of Future Population Growth and Change in Singapore” [11].

Generalised linear models with Poisson distribution and the change points [12] were performed to estimate the annual rate of asthma-related health services use (HSU) and identify periods with distinct trends. As we observed a rebound in inpatient volume during COVID-19 in the National University Health System (NUHS) -- one of the three healthcare clusters in Singapore, we excluded observations in 2020 (during the COVID-19 pandemic in Singapore) from the change-point analysis.

To project future asthma-related HSU, we developed a probabilistic model which combined the rate estimate from the latest stable trend of the change-point model (which stops at 2019) with population growth projections, the component of the model is written as: *N*(*y*) = *N*_*y*_ *exp* {*β*_0_ + *β*_1(_*y* − *y*_0_)}, where *N*(*y*) is the projected number of episodes at year y; *N*_*y*_ the projected size of Singapore’s resident population at year y; *y*_0_ the last change point identified, and *β*_0_ and *β*_1_ the intercept and coefficient for time (in years) for the logarithm of the rate of episodes, all of which were obtained from the change-point analysis in a Poisson regression framework. The joint distribution of *y*_0_, *β*_0_ and *β*_1_ were modelled via bootstrapping with 1000 iterations.

In our main analysis, we assumed constant low fertility and low migration as our base projection scenario i.e., total fertility rate (TFR) remains constant at 1.24 births per woman and 30,000 migrants are added each year throughout the projection period (Scenario 2 in “Scenarios of Future Population Growth and Change in Singapore” [11]). This base projection scenario is closest to the status quo in Singapore, where TFR is 1.04 births per woman [13] and the net migration rate in 2025-2030 is expected to be around 4.65 per 1000 population (approximately 30,000 annual net migration) [14]. In our sensitivity analysis, we reported a range of variant projections made under two additional sets of population growth assumptions: constant low fertility and closed population i.e., TFR = 1.24 births per woman with zero net migration throughout the projection period (Scenario 1 [11]), and constant low fertility and medium migration i.e., TFR = 1.24 births per woman and 60,000 migrants are added each year throughout the projection period (Scenario 3 [11]).

All costs were adjusted to 2022 USD. For asthma-related healthcare cost estimation, we followed Tan et al. in assuming that the average length of stay per asthma-related hospitalisation episode is 2 days [15] and the cost of each hospitalised day is $500 in 2013 SGD (i.e., $558.33 in 2022 SGD) [15, 16], which gives a combined estimate of SGD$1116.65 (=USD$839.59 based on 2022 USD$1 = SGD$1.33) per episode cost of asthma-related hospitalisation. We derived the average per episode asthma-related ED attendance cost, which is an estimated SGD$123 (=USD$92.48), using the average ED attendance fee from seven of the nine acute public hospitals in Singapore, including KK Women’s and Children’s Hospital (SGD$120), Tan Tock Seng Hospital (SGD$128), Singapore General Hospital (SGD$121), Changi General Hospital (SGD$126), Ng Teng Fong General Hospital (SGD$120), National University Hospital (SGD$121) and Khoo Teck Puat Hospital (SGD$122) [17].

## Results

**Fig. 1:**
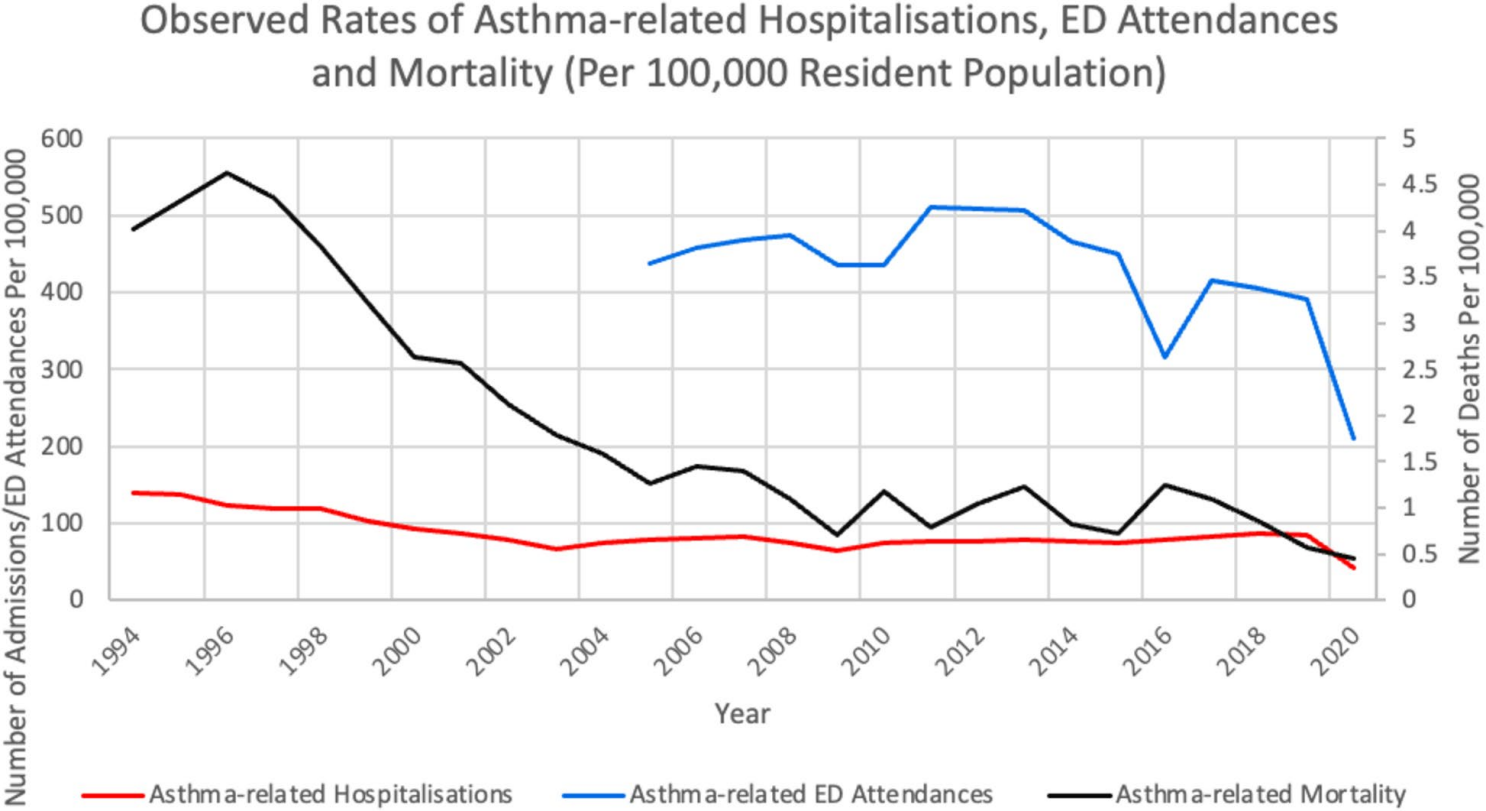
Observed Past Trends in Asthma-related Hospitalisations, ED Attendances and Mortality. Note: Describes the pattern of rate (per 100,000 resident population) observed in the past twenty-five years for asthma related admissions (in red), ED attendances (in blue) and mortality (in black) respectively

Figure 1 shows the observed past trends in asthma-related hospitalisations, ED attendances and mortality. In Fig. 1.1, we showed these observed past trends in separate panels (see Additional file 1). The rates of asthma-related hospitalisation had generally hovered between 70 and 80 episodes per 100,000 since 2003, while asthma-related ED attendance rates had mostly remained above 400 per 100,000 and recently declined to 390 in 2019; asthma mortality rates declined from 4.02 to 0.57 deaths per 100,000 between 1994 and 2019.

**Fig. 2:**
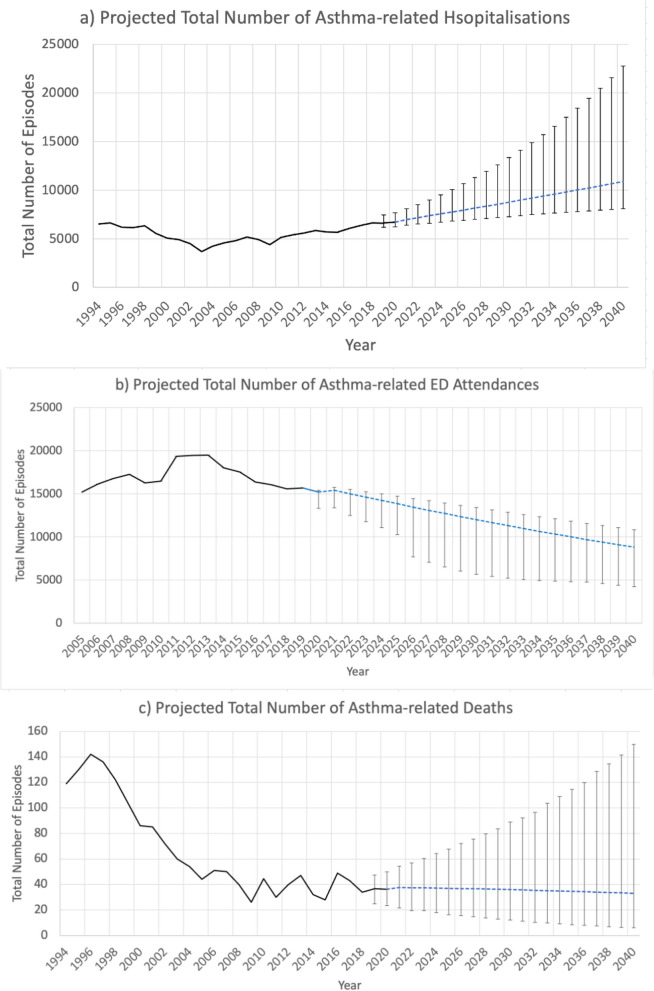
Projected Future Trends in Asthma-related Admissions, ED Attendances and Deaths (Base Projection Scenario). Legend:  Observed trend,  Projected trend (Scenario 2: TFR = 1.24 births per woman, net migration = 30,000 annual net addition),  95% credible intervals (CI) form bootstrapping regressions, Note: Panels **a**), **b**) and **c**) describe the trends in total number of episodes observed in the past (in black solid line) as well as projected trends (blue dashed lines) for asthma-related admissions, ED attendances and deaths respectively. The error bars represent the 95% credible intervals (CI) of the projections on 2019-2040

Figure 2 shows the projected future trends in asthma-related admissions, ED attendances and mortality based on the base projection scenario (Scenario 2). Asthma admissions are expected to increase by 1.7% (95% CI: 0.7 to 5.0%) per year, amounting to an estimated 163,633 total episodes between 2023 and 2040. There is a tendency for asthma-related ED attendances to decrease over time (change per year: -3.4, 95% CI: − 5.8 to 0.0%), which will lead to an estimated total of 208,145 asthma-related ED attendances between 2023 and 2040. On the other hand, the asthma mortality rate is likely to remain stably low (change per year: -1.3, 95% CI: − 11.0 to 4.3%) with an estimated 638 total episodes of asthma-related deaths between 2023 and 2040.

**Fig. 3:**
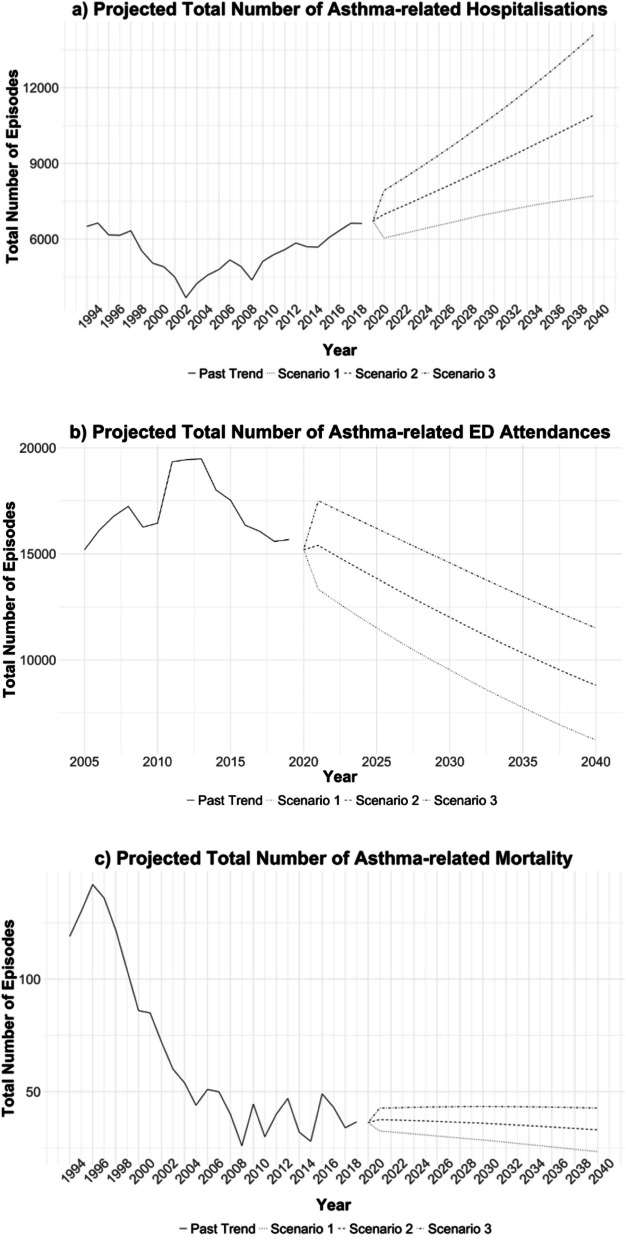
Projected Future Trends in Asthma-related Admissions, ED Attendances and Deaths (Various Projection Scenarios). Legend:  Observed trend,  Projected trend (Scenario 1: TFR = 1.24 births per woman, zero net migration),  Projected trend (Scenario 2: TFR = 1.24 births per woman, net migration = 30,000 annual net addition),  Projected trend (Scenario 3: TFR = 1.24 births per woman, net migration = 60,000 annual net addition), Note: Panels **a**), **b**) and **c**) describe the trends in total number of episodes observed in the past (in black solid line) as well as the projected trends under Scenario 1 (dashed lines), Scenario 2 (dashed lines) and Scenario 3 (dot-dashed lines) for asthma-related admissions, ED attendances and deaths respectively

Figure 3 shows our projection results from sensitivity analyses performed based on various projection scenarios. Between 2023 and 2040, the projected total number of asthma admissions is 163,633 in the base projection scenario (TFR = 1.24 births per woman, net migration = 30,000 net annual addition), which ranges from 126,723 (Scenario 1: TFR = 1.24 births per woman, zero net migration) to 200,421episodes (Scenario 3: TFR = 1.24 births per woman, net migration = 60,000 net annual addition); the projected total number of asthma-related ED attendances is 208,145 in the base projection scenario, ranging from 163,616 (Scenario 1) to 254,160 episodes (Scenario 3); the projected total number of asthma-related deaths is 638 in the base projection scenario, ranging from 498 (Scenario 1) to 777 episodes (Scenario 3).

## Discussion

Based on our findings, changes in asthma-related ED attendance rate were more dynamic overtime and thus unstable, whereas the trend of asthma-related hospitalisations had been stable. A possible explanation was that a considerable proportion of ED attendance was attributed by non-essential health seeking behaviour, which was more susceptible to the influences of interventions or policy changes albeit the effect was more temporarily. In fact, findings from a previous study suggest that a large proportion of ED visits by asthmatic patients who are frequent ED-attenders do not necessarily require emergency care [18]. Besides, trend analysis performed on asthma-related ED attendances was based on 15-year aggregate-level annual data, whereas we had 25 years of data on asthma-related hospitalisations and mortality.

Compared to other OECD countries, while asthma-related mortality in Singapore was much lower [19, 20], the estimated asthma admission rate was approximately twice the OECD average [4]. The current asthma-related ED attendance rate was 20% lower than that in the US [21] but five times that in the UK [22]. These observations indicate a high burden of asthma acute care, consistent with recent evidence on excess asthma-related resource use [18] and gaps in asthma primary care in Singapore [23].

In addition, we observed a sharp decline in asthma-related HSU during the COVID-19 pandemic, whereas asthma-related deaths were rarely affected. Between 2019 and 2020, asthma admission and ED attendance respectively declined from 84 to 41, and from 390 to 211 per 100,000 (51.1 and 46.0% decrease), consistent with the trends observed during previous global respiratory pandemics i.e., SARS in 2003 and H1N1 in 2009. Factors such as patients’ fear of nosocomial transmissions, and COVID-19 measures such as universal masking and social distancing which may have led to a reduction in respiratory infections that might trigger an asthma attack [24] are potential reasons for the observed reduction in asthma-related acute care during the COVID-19 pandemic in Singapore. Moreover, in the same period, we observed an increase in dispensation ratio of preventer medication (in particular, inhaled corticosteroids) over reliever medication, which we found to be associated with significantly reduced asthma-related urgent and emergent care [25]. Of note, we also observed that following the outbreak of SARS and H1N1, there were quick rebounds subsequently in the corresponding volumes of HSU, consistent with findings from previous studies [26] and data from NUHS.

Between 2023 and 2040, the projected total number of asthma admissions corresponds to a total of USD$103,075,820 in the base projection scenario, ranging from USD$80,498,374 (Scenario 1) to USD$125,569,156 (Scenario 3). However, there is potential room for Singapore to reduce its asthma admission rate, considering that the OECD average is approximately 40 per 100,000 per year [27]. The OECD average is used as a benchmark of comparison as most OECD countries, alike Singapore, have achieved universal health coverage [28]. If Singapore could also keep its asthma admission rate below 40 per 100,000 per year, the society might save USD$ 77,291,781 between 2023 and 2040 i.e., a 75% cost reduction (similar percentages of cost reduction across all projection scenarios). Our projections also show that between 2023 and 2040, the projected total number of asthma-related ED attendances corresponds to USD$15,053,795 in the base projection scenario, ranging from USD$11,927,995 (Scenario 1) to USD$18,278,483 (Scenario 3).

Nonetheless, recent developments in Singapore’s healthcare system may positively impact asthma management, thereby potentially reducing avoidable admissions and costs. For instance, in its early years of establishment, the Singapore National Asthma Programme (SNAP) had strived to enhance the regular use of inhaled corticosteroids nationwide [8]. With their initial success [8], and recognising the current gaps in asthma care [23], since 2022, SNAP has extended its goals to reinforce regular follow-up of asthma patients in primary care [29]. Concurrently, the launch of another nationwide initiative -- “Healthier SG” complements the SNAP efforts by strengthening patient-general practitioner (GP) ties through the “one family one doctor” regime [30]. The effect of such asthma primary care reinforcement on the reduction of asthma-related tertiary care burden and associated costs had been proven in other settings [31, 32]. The Finnish asthma programme which focused on early detection, delivery of anti-inflammatory treatment, self-management and good networking with GPs [31, 33] had led to a 50% reduction in asthma hospitalisation days, and a 24 and 61% reduction in asthma ED visits in adults and children respectively [34]. Another instance is the Polish pilot asthma programme [31, 35], which had similar scope as the Finnish programme (i.e., focusing on early diagnosis and the role of GPs) [31, 35] and had resulted in a 43% reduction in asthma admissions and a simultaneous reduction in hospitalisation length by 2.5 days.

This study has several imitations. First, based on aggregate-level HSU and mortality data, our work only provides a description of the healthcare landscape in Singapore; without patient-level data, in-depth analyses which account for intrinsic factors (e.g., medication use, comorbidity etc.) or subgroup variations could not be performed. Second, we did not adjust for extrinsic factors that may affect these HSU trends, such as air pollution and changes in treatment approach. Third, the wide 95% credible intervals in our reported projections was another limitation which was potentially related to the high fluctuations of aggregated, annually-measured HSU and mortality rates overtime, which could vary substantially under policy and pandemic influences. To reduce such uncertainty in our projections, in our future analysis, we will use patient-level data to collect monthly measurements, and estimate and combine the projections form relatively homogeneous sub-populations, such as from age- and sex-stratified trend projections. In addition, to account for comorbidity-attributable burden while controlling for general non-essential healthcare utilisation, we will estimate the incremental healthcare burden of asthma patients over and beyond that of the non-asthma general population. We will also develop simulation models to predict the impact of reinforced primary care through SNAP and Healthier SG on the reduction of preventable tertiary care burdens including non-essential asthma-related ED attendance.

## Conclusions

Our work addressed the lack of up-to-date evidence on population-wide burden of asthma in Singapore. Our findings indicate that current rates of asthma-related hospitalisations and ED attendances in Singapore are roughly twice of the average of other OECD countries. While asthma-related ED visits and mortality have stabilised, asthma admissions will increase over the next 20 years in Singapore. The existing national asthma programme in Singapore, together with the recent efforts in reinforcing primary care at the national level, provides opportunities to reduce avoidable asthma admissions.

### Supplementary Information

**Additional file 1: Figure 1.1. **Observed Rates of Asthma-related Hospitalisations, ED Attendances and Mortality Per 100,000 Resident Population (in separate panels). Note: Panels a), b) and c) describes the pattern of rate (per 100,000 resident population) observed in the past twenty-five years for asthma related admissions, ED attendances and mortality respectively.

## Data Availability

The datasets generated and/or analysed during the current study are not publicly available but are available from the corresponding author on reasonable request.

## References

[1] Vos T, Lim SS, Abbafati C, Abbas KM, Abbasi M, Abbasifard M, Abbasi-Kangevari M, Abbastabar H, Abd-Allah F, Abdelalim A, Abdollahi M (2020). Global burden of 369 diseases and injuries in 204 countries and territories, 1990–2019: a systematic analysis for the global burden of disease study 2019. Lancet..

[2] Asher MI, Rutter CE, Bissell K, Chiang CY, El Sony A, Ellwood E, Ellwood P, García-Marcos L, Marks GB, Morales E, Mortimer K (2021). Worldwide trends in the burden of asthma symptoms in school-aged children: global asthma network phase I cross-sectional study. Lancet..

[3] Bahadori K, Doyle-Waters MM, Marra C, Lynd L, Alasaly K, Swiston J, FitzGerald JM (2009). Economic burden of asthma: a systematic review. BMC Pulm Med..

[4] OECD Library: Health at a Glance 2021, OECD indicators. https://www.oecd-ilibrary.org. Accessed 20 Jul 2023.

[5] Global Initiative for Asthma: Global strategy for asthma management and prevention, updated 2017. Available on https://ginasthma.org (2023). Accessed 29 Jul 2023.

[6] Ministry of Health Singapore: Singapore Burden of Disease Study 2010. https://www.moh.gov.sg/resources-statistics/reports/singapore-burden-of-disease-study-2010 (2018). Accessed 19 Nov 2022.

[7] Finkelstein EA, Lau E, Doble B, Ong B, Koh MS (2021). Economic burden of asthma in Singapore. BMJ Open Respir Res..

[8] Chong PN, Tan NC, Lim TK (2008). Impact of the Singapore National Asthma Program (SNAP) on preventor-reliever prescription ratio in polyclinics. Ann Acad Med Singap..

[9] Ng TP, Niti M, Tan WC (2003). Trends and ethnic differences in asthma hospitalization rates in Singapore, 1991 to 1998. Ann Allergy Asthma Immunol..

[10] Department of Statistics Singapore: Population and population structure. https://www.singstat.gov.sg/find-data/search-by-theme/population/population-and-population-structure/latest-data (2022). Accessed 23 Sep 2022.

[11] Institute of Policy Studies: IPS Report on “Scenarios of Future Population Growth and Change in Singapore”. https://lkyspp.nus.edu.sg/ips/research/governance-of-a-city-state/ips-report-on-scenarios-of-future-population-growth-and-change-in-singapore (2011). Accessed 23 Sep 2022.

[12] Muggeo VMR (2003). Estimating regression models with unknown break-points. Stat Med..

[13] Department of Statistics Singapore: Births and Fertility. https://www.singstat.gov.sg/find-data/search-by-theme/population/births-and-fertility/latest-data (2023). Accessed 1 Nov 2023.

[14] Unicef: Singapore Migration Profiles. https://esa.un.org/miggmgprofiles/indicators/files/Singapore.pdf. Accessed 1 Nov 2023.

[15] Tan NC, Nguyen HV, Lye WK, Sankari U, Nadkarni NV (2016). Trends and predictors of asthma costs: results from a 10-year longitudinal study. Eur Respir J..

[16] Singapore Ministry of Health: hospital bill sizes: asthma. www.moh.gov.sg/content/moh_web/home/costs_and_financing/HospitalBillSize/asthma.html. Accessed 28 Dec 2014.

[17] Teng A (2022). 24-hour emergency fees in Singapore a&E, clinics and ambulance.

[18] Lim SF, Wah W, Pasupathi Y, Yap S, Koh MS, Tan KL, Chay CJ, Ong ME (2014). Frequent attenders to the ED: patients who present with repeated asthma exacerbations. Am J Emerg Med..

[19] Centers for Disease Control and Prevention: Most Recent National Asthma Data. https://www.cdc.gov/asthma/most_recent_national_asthma_data.htm (2023). Accessed 3 Dec 2022.

[20] Australian Institute of Health and Welfare. Asthma. https://www.aihw.gov.au/reports/chronic-respiratory-conditions/asthma-1 (2023). Accessed 29 Jul 2023.

[21] Centre for Disease Control and Prevention: Asthma emergency department (ED) visits 2010–2018. https://www.cdc.gov/asthma/asthma_stats/asthma-ed-visits_2010-2018.html. Accessed 1 Oct 2023.

[22] Nuffieldtrust: Potentially Preventable Emergency Admissions. https://www.nuffieldtrust.org.uk/resource/potentially-preventable-emergency-hospital-admissions. Accessed 1 Oct 2023.

[23] Yii A, Puah SH, Lim HF, Tay TR, Neo LP, Li A, Lau P, Tan RA, Tan LL, Abisheganaden J, Lim TK (2018). A national audit of severe life-threatening asthma in Singapore. Eur Respir J..

[24] Wee LE, Conceicao EP, Tan JY, Sim JX, Venkatachalam I (2021). Reduction in asthma admissions during the COVID-19 pandemic: consequence of public health measures in Singapore. Eur Respir J..

[25] Lim LHM, Lim HF, Liew MF, Chen W (2023). Impact of guideline-based asthma treatment on health services use in Singapore before and during COVID-19 outbreak. J Asthma Allergy..

[26] Chu D, Chen RC, Ku CY, Chou P (2008). The impact of SARS on hospital performance. BMC Health Serv Res..

[27] Australian Institute of Health and Welfare: OECD Health Care Quality and Outcome Indicators, Australia 2021. https://www.aihw.gov.au/reports/international-comparisons/oecd-health-care-quality-and-outcomes-indicators-2021/contents/primary-care-avoidable-hospital-admissions (2022). Accessed on 20 Jul 2023.

[28] Sasaki T, Izawa M, Okada Y (2015). Current trends in health insurance systems: OECD countries vs. Japan Neurol Med Chir..

[29] Agency for Integrated Care: Singapore National Asthma Programme (SNAP). https://www.primarycarepages.sg/schemes-and-programmes/singapore-national-asthma-programme (2019). Accessed 20 Jul 2023.

[30] Healthier SG: White Paper on Healthier SG. https://www.healthiersg.gov.sg/resources/white-paper/ (2023). Accessed 29 Jul 2023.

[31] Kupczyk M, Haahtela AA, Kuna CP (2010). Reduction of asthma burden is possible through National Asthma Plans. Allergy..

[32] Kringos DS, Boerma W, van der Zee J, Groenewegen P (2013). Europe’s strong primary care systems are linked to better population health but also to higher health spending. Health Aff..

[33] Haahtela T, Laitinen LA (1996). Asthma programme in Finland 1994–2004. Report of a working group. Clin Exp Allergy..

[34] Haahtela T, Tuomisto LE, Pietinalho A, Klaukka T, Erhola M, Kaila M, Nieminen MM, Kontula E, Laitinen LA (2006). A 10 year asthma programme in Finland: major change for the better. Thorax..

[35] Stelmach W, Majak P, Jerzynska J, Stelmach I (2005). Early effects of asthma prevention program on asthma diagnosis and hospitalization in urban population of Poland. Allergy..

