# Supplementary material for: The increasing burden of asthma acute care in Singapore: an update on 15-year population-level evidence
Source: BMC Pulm Med. 2023 Dec 12;23:502. doi: 10.1186/s12890-023-02797-7 (PMC10714448; doi:10.1186/s12890-023-02797-7)
Supplement: Supplementary file 1 — Additional file 1: Figure 1.1. Observed Rates of Asthma-related Hospitalisations, ED Attendances and Mortality Per 100,000 Resident Population (in separate panels). Note: Panels a), b) and c) describes the pattern of rate (per 100,000 resident population) observed in the past twenty-five years for asthma related admissions, ED attendances and mortality respectively. [file 12890_2023_2797_MOESM1_ESM.pdf]

Appendix 1

Figure 1.1: Observed Rates of Asthma-related Hospitalisations, ED Attendances and Mortality Per 100,000 Resident Population (in separate panels)

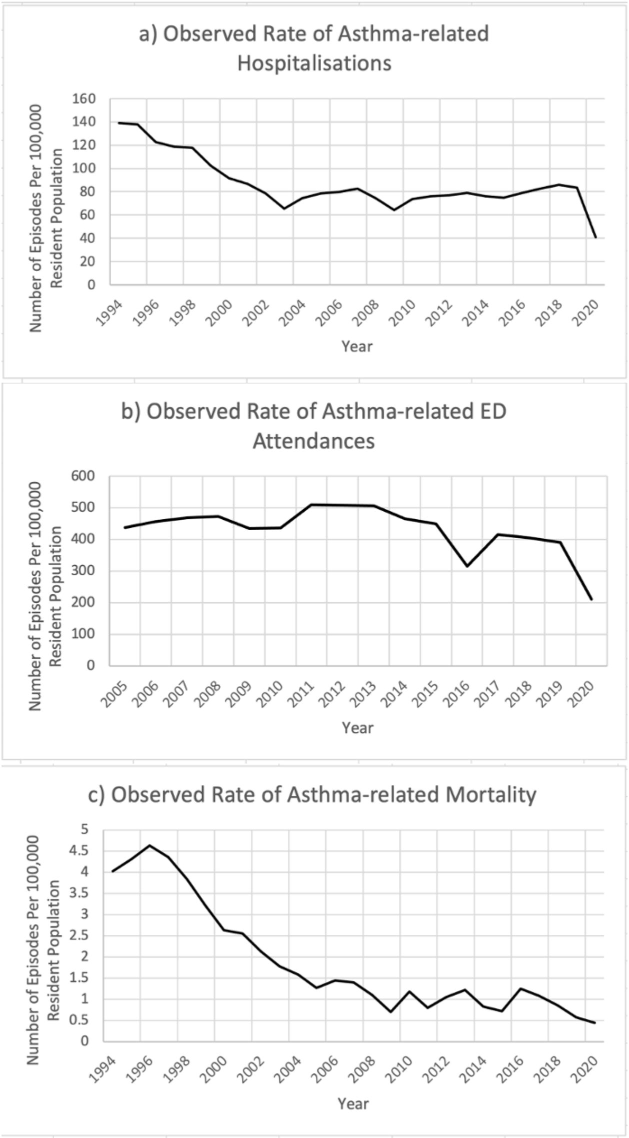

Note: Panels a), b) and c) describes the pattern of rate (per 100,000 resident population) observed in the past twenty-five years for asthma related admissions, ED attendances and mortality respectively.
